# Cigarette butt waste as material for phase inverted cellulose acetate membranes synergistically incorporated with silver/copper oxide (CA-Ag/CuO) for reverse osmosis water treatment

**DOI:** 10.1039/d6ra00485g

**Published:** 2026-02-27

**Authors:** Livhuwani Mafhala, Nomcebo Khumalo, Shohreh Azizi, Ilunga Kamika

**Affiliations:** a UNESCO-UNISA Africa Chair in Nanoscience and Nanotechnology College of Graduates Studies, University of South Africa Muckleneuk Ridge Pretoria 392 South Africa azizis@unisa.ac.za; b Department of Environmental Sciences, College of Agriculture and Environmental Sciences, University of South Africa Private Bag X06 Florida 1710 South Africa; c Nanotechnology and Water Sustainability Research Unit, College of Science, Engineering, and Technology, University of South Africa Florida Johannesburg 1709 South Africa

## Abstract

The improper disposal of cigarette butts presents a significant environmental challenge, yet their cellulose acetate (CA) content offers the potential for upcycling into functional materials. In the study, the potential of using CA waste obtained from cigarette butts for fabricating CA phase-inverted membranes is demonstrated. The CA membranes were impregnated with silver (Ag) and copper oxide nanoparticles (CuO) simultaneously to improve permeation flux and salt rejection during reverse osmosis. The effect of the variation of Ag/CuO on the CA membranes was probed using Fourier transform infrared spectroscopy (FTIR) which confirmed the successful introduction of Ag/CuO NPs into the CA matrix. The effect of the nanoparticles on the membrane morphology was assessed using scanning electron microscopy (SEM). An increase in the contact angle measurements concurrent with higher nanoparticle loading was observed, indicating an improved membrane hydrophilicity. Membrane performance was evaluated using a crossflow RO test whereby a significant increase in pure water flux (up to 73.33 Lm^−2^ h^−1^ for CA_0.15 (Ag/CuO)) and effective desalination of a 30 000 ppm NaCl solution under 4 bar pressure was observed. The rejection of NaCl was 92%, alongside a flux recovery ratio (FRR) of 94% at the optimal nanoparticle concentration. Additionally, the nanocomposite membranes demonstrated strong anti-scaling behavior, as evidenced by the reduced irreversible fouling and improved FRR. Overall, the current study highlights the dual benefits of environmental waste valorization, offering a viable, low-cost fabrication alternative to conventional membranes. Furthermore, the use of bio-based CA ensures easier biodegradation compared to other polymers. Consequently, the cigarette butt-derived CA-Ag/CuO membranes present an effective and environmentally suitable solution for saline water desalination, particularly in rural areas.

## Abstract 2

The improper disposal of cigarette butts presents a significant environmental challenge, yet their cellulose acetate (CA) content offers the potential for upcycling into functional materials.

## Graphical abstract

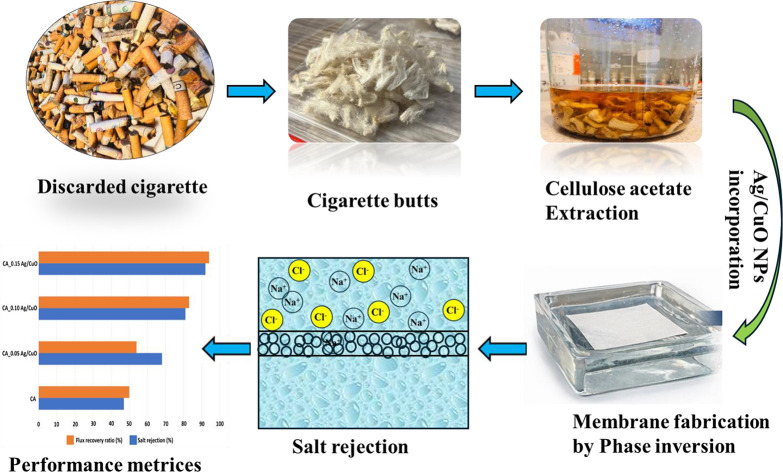

## 1 Introduction

Global water scarcity, exacerbated by climate change and failing water infrastructure, has forced many South African communities to rely on untreated groundwater and brine.^1,2^ This vulnerability was optimized by Cape Town's “Day zero” crisis and remains a daily reality in regions like Limpopo, where borehole water is often consumed without purification.^3^ Chronic reliance on such untreated sources poses significant health risks, for instance, high concentrations of dissolved fluoride and minerals in groundwater are linked to dental fluorosis, leading to enamel decay and tooth discoloration.^4,5^

Addressing these challenges requires a shift toward decentralized, point-of use treatment systems that can be integrated with existing borehole infrastructure. Desalination technologies, particularly reverse osmosis (RO), have emerged as a promising route to supplement existing water resources by tapping into unconventional water sources, such as seawater, brackish water, and saline groundwater.^6^ RO is particularly advantageous because of its high salt rejection, relatively low energy consumption compared with thermal methods, and adaptability to modular installations suitable for remote areas.^7^

Among other materials used for fabrication of RO membranes, cellulose acetate (CA) is notable because of its biocompatibility, moderate hydrophilicity, and good film-forming properties. Interestingly, CA is also the primary component of cigarette filters, which are typically discarded as waste and constitute major environmental pollutants. The reuse of cigarette butts as a source of CA not only provides sustainable feedstock for membrane fabrication but also contributes to waste valorization and environmental remediation.^8^ Several strategies have been investigated in the context of cigarette butts (CBs), including reports on cellulose acetate polymers to obtain carbon-based materials for gas adsorption, sensors, catalysis, energy applications,^9–15^ and oil-water separation.^16–19^

Moreover, previous research has successfully converted cellulose acetate from CBs into loose nanofiltration membranes for selective separation (dyes and monovalent and divalent salts).^20^ Furthermore, the efficiency of cellulose acetate extracted from CBs was investigated for the removal of heavy metals (cadmium, chromium, and lead) from aqueous solutions in a forward osmosis reactor.^21^ However, the microfibrous nature of CBs can eventually lead to leaching during the separation process. To overcome this obstacle, Liu *et al.* (2019) fabricated stainless-steel mesh-coated nanofibrous CA membranes *via* electrospinning for oil/water separation.^22^ Several studies have explored the use of CA-based membranes in reverse osmosis systems. For example, Sagle and Freeman (2004) reviewed the role of CA in early RO membranes, emphasizing its good separation properties and processability. More recent studies have focused on improving the performance of CA membranes by blending them with nanomaterials.^23^ Heidari *et al.* (2023) embedded graphene oxide-poly (amidoamine) dendrimer nanocomposites into CA membranes for enhanced salt rejection.^24^ Nevertheless, very few studies have explored the dual use of recycled cigarette butt-derived CA and green-synthesized nanomaterials.

This study builds upon this gap by incorporating Ag/CuO nanoparticles previously synthesized from agricultural waste^25,26^ into phase-inverted CA membranes derived from recycled cigarette butts, specifically aimed at reverse osmosis of saline or brackish water. The rationale for the synergistic incorporation of dual nanoparticles into the polymer matric lies in the pursuit of multifunctional performance. While silver (Ag) provides superior broad-spectrum antimicrobial properties to combat biofouling, copper oxide (CuO) enhances the membrane's hydrophilicity and mechanical stability, collectively overcoming the inherent trade-offs between permeability and selectivity in cellulose acetate membranes. The combination of waste valorization (cigarette filters and fruit peels) and nanotechnology offers a sustainable and eco-friendly approach to synthesize more RO membrane materials. This work not only extends the existing research on CA-based membranes but also introduces a novel, integrated waste-reuse strategy that addresses both environmental pollution and water purification needs.

## 2 Materials and methods

### 2.1 Reagents and chemicals

Cellulose acetate (CA) was extracted from discarded cigarette butts (CBs) collected around Gauteng (Johannesburg) and Cape Town public smoking places for the fabrication of CA-based membranes. Analytical grade *N*-methyl-2-pyrrolidone (NMP) was purchased from the monitoring and control laboratories (PTY). Previously synthesized Ag and CuO^25,26^ nanoparticles were used to modify the CA membranes. Sodium chloride (NaCl) reagentplus®,99% was purchased from Sigma-Aldrich. Ethanol (99.9%) was procured from CCIMELMANN (PTY) Ltd. Finally, deionized water obtained from Milli-Q system (Millipore, USA) water was also used.

### 2.2 Cellulose acetate extraction from used cigarette butts (CBs)

During the fabrication of the CA-based membrane, the collected CBs were washed according to the method described by De Fenzo *et al.* (2020) with slight modifications.^27^ Briefly, wrapping paper, tobacco, and burning tips were removed. Subsequently, the CBs were washed in hot water (50 °C) for 60 min while stirring at 400 rpm. The CBs were washed thrice with cold water. To remove potential organic compounds, the butts were twice washed in ethanol (99.9%) after which they were dried at 30 °C overnight in an air-circulating oven to remove the moisture content. To assess the washing method, SEM was used on both the washed and unwashed butts to determine the presence of surface-adhered substances.

### 2.3 Membrane preparation

For CA-based membrane preparation, the dope was prepared by dispersing 18 wt% of the cleaned CB filters in a corresponding amount of *N*-methyl-2-pyrrolidone (80 wt%) without any additives. A homogeneous mixture was obtained after stirring the mixture for 24 h at 50 °C. Prior to membrane casting, the solution was degassed for 24 h in dark cardboard to remove trapped air bubbles. The membrane was then cast on a glass plate using a casting knife to make a flat membrane with an optimized thickness of 0.15 ± 0.06 mm prior to phase inversion obtained by immersion in a distilled water coagulation bath overnight.

### 2.4 Synergistic incorporation of Ag/CuO nanoparticles into CA polymer

CA-Ag/CuO nanocomposite membranes were individually fabricated *via* the phase inversion technique with a blend composition of 18 wt% by varying the concentration of CuO^25^ and Ag^26^ nanoparticles (0.05, 0.10 and 0.15 wt%) at a 1 : 1 ratio in the NMP solvent (82 wt%), as shown in Table 1. First, the Ag/CuO NPs were added to the NMP solvent and ultrasonicated for 20 min before dissolving the polymer (doping) to obtain a fine dispersion of the NPs in the solvent. Subsequently, the polymer was then dissolved and stirred for 24 h at 50 °C to obtain a homogenous dope solution. The obtained dope was degassed to remove air bubbles by placing the dope solution in a dark carboard for 24 h prior to casting. Using a casting knife from electrometer, the membranes were cast on a glass plate to make a flat membrane with an optimized thickness of 0.15 ± 0.06 mm. The cast membranes were exposed to air for 30 s before immersing the glass plate in a coagulation bath. The membranes were stored in water and dried prior to characterization.

**Table 1:** Composition of pristine CA and CA-Ag/CuO nanocomposite membranes

Membrane	Polymer (CA) (wt%)	Solvent (NMP) (wt%)	1 : 1 ratio of Ag/CuO NPs (wt%)
CA	18	82	0
CA _0.05 (Ag/CuO)	18	82	0.05
CA_0.10 (Ag/CuO)	18	82	0.10
CA_0.15 (Ag/CuO)	18	82	0.15

### 2.5 Membrane characterization

Scanning electron microscope (SEM) (ZEISS Crossbeam with GEMINI 340 FIB-SEM) was used to study the membrane morphology which includes morphology of the membrane pores and uniformity on the surface of the membrane and cross section. Prior to SEM analysis, the membrane samples were dipped and fractured in liquid nitrogen and then sputtered with gold to prevent charge build up by creating a conductive surface to enhance image quality. To determine the surface functional groups of the fabricated membranes, Fourier Transform Infrared (FTIR) spectroscopy was employed. The measurements were conducted using a PerkinElmer Frontier Optica FTIR spectrometer (Massachusetts, USA) across the mid-infrared range 4000–400 cm^−1^. Additionally, membrane surface analysis was performed using a combination of techniques. A confocal laser scanning microscope (CLSM) (Carl Zeiss LSM 900) and an atomic force microscope (AFM) (Witec Alpha 300 A (TS-150)) were employed for detailed surface characterization (GmbH, Germany). Subsequently, a Drop Shape Analyzer (DSA3OE Kruss) measured the membrane's contact angle.

### 2.6 Membrane performance test

#### 2.6.1 Pure water flux

The pure water flux (PWF) of the fabricated membranes was measured using a cross-flow reverse osmosis system with an effective membrane area of 42 cm^2^. Initially, the membranes were compacted for 30 minutes at a transmembrane pressure of 3 bar to regulate and stabilize the membrane flux. The permeation flux of the fabricated membranes was measured at four bars using eqn (1). The reverse osmosis time was 30 min in all experiments.1
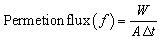
where *W* is the quantity of permeate collected (V), *F* is the water permeability (in Lm^−2^ h^−1^), *A* is the effective membrane area (m^2^), and Δ*t* is the reverse osmosis time (h).

#### 2.6.2 Salt (NaCl) rejection

Sodium chloride (0.5 M) was then prepared. Each membrane was subjected to a NaCl rejection study, and the percentage rejection was evaluated using eqn (2).2
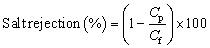
where *C*_p_ and *C*_f_ represent the concentrations of the permeate and feed solution, respectively.

#### 2.6.3 Flux recovery ratio, irreversible, and reversible fouling

The fouling resistances of all fabricated membranes were measured using the flux recovery ratio (FRR), reversible and irreversible fouling. After the NaCl rejection study was completed, the membranes were cleaned by sonicating the membranes in deionized water for 30 min at room temperature, and then the water flux was measured (pure water permeation flux) *F*_1_ and salt water was designated as *F*_2_ and calculated using eqn (3).3
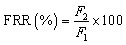


During the reverse osmosis, salt ions diffused through the pores of the membrane, causing membrane scaling.^28^ The high NaCl concentration can cause the polymer matrix to swell which was evaluated by eqn (2) after membrane cleaning. However, the trapped NaCl ions on the surface of the membranes caused reversible fouling, which was easily removed by membrane cleaning *via* sonication and was evaluated using eqn (5) (ref. 29) which represents the pure water flux after washing the membranes.4
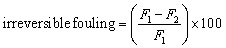
5
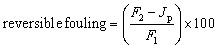


## 3 Results and discussion

**Fig. 1:**
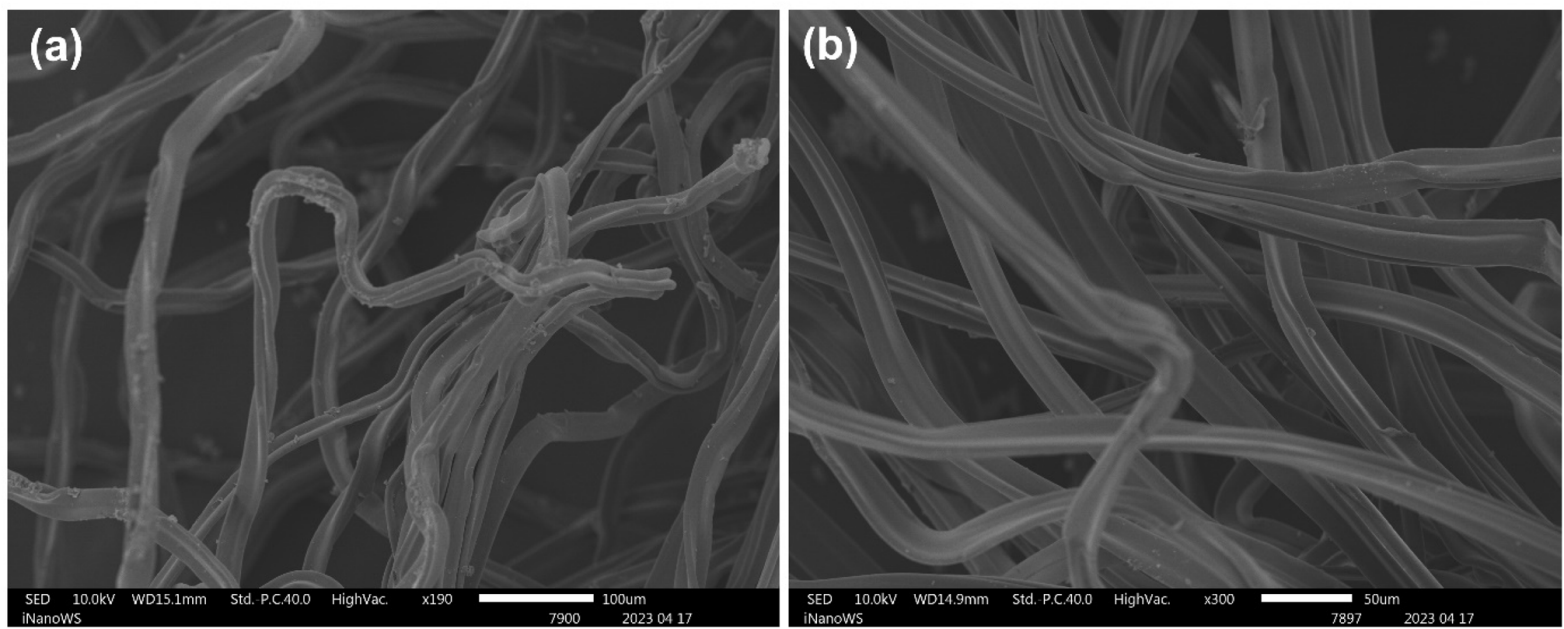
Scanning electron microscopy images of used cellulose acetate fibers (a) smoked unwashed cellulose acetate fibers at 50 µm magnification showing particles adhered on the surface of the fibers (b) smoked washed cellulose acetate fibers at 100 µm magnification with no adhered particles on the surface of the fibers.

In this study, cigarette butts were collected to extract the cellulose acetate polymer for potential use in the fabrication of reverse osmosis membranes for wastewater treatment. The extracted cellulose acetate was thoroughly washed to eliminate adhered particles, and the effectiveness of the washing process was evaluated using SEM (Fig. 1). Wherein, microscopic images of smoked unwashed CA and smoked CA recovered (washed) from CB samples are presented in Fig. 1a and b, respectively. It was observed that there are suspended particles on the surface of the fibers in Fig. 1a. However, after washing, the surfaces of the fibers were smooth, indicating that no particles adhered to the CA fibers (Fig. 1b). This shows that the washing method was effective in removing almost all contaminants that adhered to the surface of the fibers. After using the same cleaning methods, De Fenzo *et al.* (2020) reported that the volume of the constituent fibers of the specimens was reduced by the cleaning process. Furthermore, an irregular surface was observed after cleaning the CA fibers^27^ which was not the case in this study. However, herein, an irregular surface was observed in both washed and unwashed fibers.

**Fig. 2:**
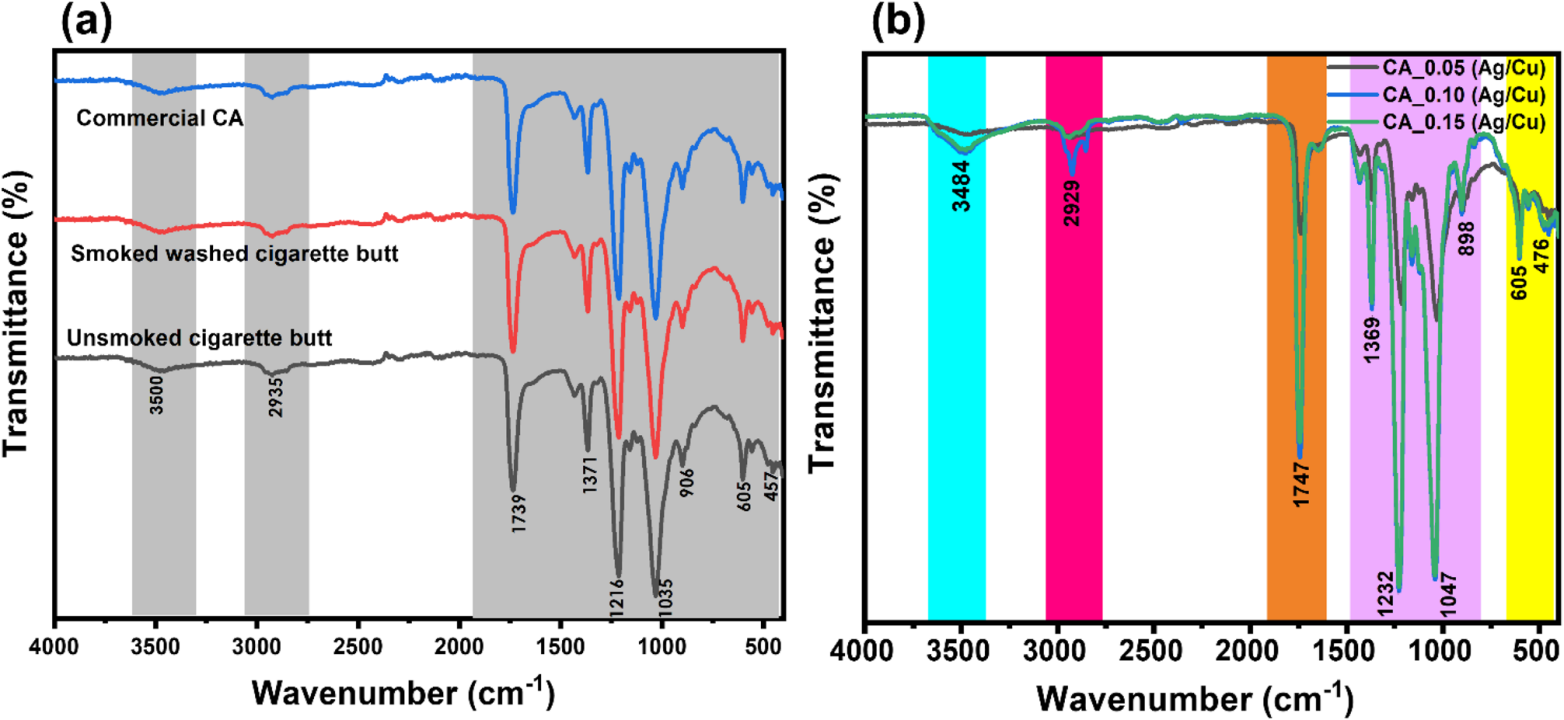
Fourier transform infrared spectroscopy spectra of (a) cellulose acetate membrane, smoked-washed cigarette butt, and unsmoked cigarette butt, (b) cellulose acetate membranes embedded with different concentrations of nanoparticles.

The molecular structures of CA obtained from CBs and commercial CA were analyzed using FTIR (Fig. 2a). The FTIR spectra of commercial CA, smoked washed CB-derived CA, and unsmoked CB-derived CA demonstrated that all samples possessed comparable chemical functionalities. They all show major absorption peaks at 1739 cm^−1^, 1216 cm^−1^, and 1035 cm^−1^ which correspond to the stretching of CA, symmetric, and asymmetric stretching vibrations (C=O carbonyl stretching, C–O stretching of acetyl groups, and CO–O–CO stretching), respectively. The small absorption band at 2935 cm^−1^ corresponds to the stretching of C–H, which are aliphatic groups, and that at 1371 cm^−1^ corresponds to hydroxyl groups and C–O groups, respectively.^30^ Additionally, there is a broad undefined peak around 3500 cm^−1^ which is attributed to the stretching vibration of the O–H group. Doyan *et al.* 2021 and Liu *et al.* 2019 reported comparable findings for CA phase-inverted membranes for oil/water emulsion separation and CA filters as nanofibrous membranes for on-demand immiscible oil/water mixtures and emulsion separation, respectively.^22,31^ Andrade *et al.* 2021 also reported similar peaks for commercial CA. The spectrum depicted in Fig. 2a is similar to that observed by Ang *et al.* (2021) for a phase-inverted membrane made from a commercial CA polymer with minor impurities. The accumulation of tobacco smoke condensates was visually evident by the light brown discoloration of the polymers suggesting that the cleaning protocol was insufficient to fully desorb the tar and oxidized nicotine trapped within the cellulose acetate fiber matrix. These contaminants may alter the characteristics of the resulting membranes.^31^ Nevertheless, this proves the effective harvesting of cellulose acetate from the discarded cigarette butt and is compatible with the unsmoked cigarette butt, and it showed successful synthesis of the CA-based membrane. Fig. 2b shows the functional groups of the CA membranes incorporated with Ag/CuO NPs. The spectra of the modified or mixed-matrix membranes resembled those of pristine CA, with no significant changes in the peaks. The functional groups of the incorporated NPs were detected, and the peaks varied with the concentration incorporated. The properties of the incorporate nanoparticles are summarized in Table 2.

**Table 2:** Characteristics of the incorporated green synthesized Ag and CuO nanoparticles

	NPs size (nm)	Absorption wavelength (nm)	Active functional groups	Zeta potential (mV)	Green synthesis material	References
Silver nanoparticles (AgNPs)	23	424	O–H; C=O; C–H	−24.6	*Citrus unshiu* fruit peel	26
Copper oxide nanoparticles (CuONPs)	32.5	260	O–H; N–H; C–N; C–O; Cu–O	+10.45	*Citrus unshiu* fruit peel	25

**Fig. 3:**
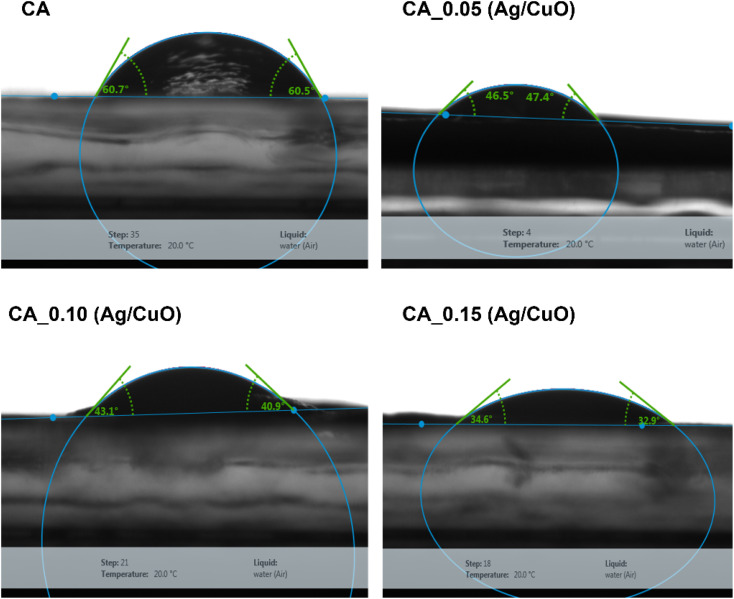
Representative images of DI water droplets for contact angles analysis on the surface of fabricated membranes. Measurements were carried out at room temperature (20 °C), without the addition of ionic strength, and at unadjusted pH.

The water contact angle is frequently used to assess the hydrophilicity of a membrane and has a significant impact on the membrane permeation rate. The contact angles of the fabricated membranes are shown in Fig. 3. As shown in Fig. 3, the water contact angle of CA, CA_0.05 (Ag/CuO), CA_0.10 (Ag/CuO), CA_0.15 (Ag/CuO) are 60.7°, 46.5°, 43.1°, and 34.6° respectively. Fig. 3 shows that the pristine CA membrane had a contact angle of 60.7° while the membranes incorporated with Ag/CuO Nps had a smaller contact angle. The gradual decline in the contact angle indicates improved hydrophilicity of the membranes owing to the incorporation of negatively charged Ag/CuO NPs on the membrane surface.^32^ It was observed that as the concentration of the NPs increased, the contact angle decreased. During phase inversion, nanoparticles, negative charge and oxygen-containing groups of the membrane interact strongly with water, which leads to higher hydrophilicity and a lower measured contact angle.^33^ This accounts for the decrease in contact angle as nanoparticle concentration increases. Similar results were reported after the incorporation of AgNPs onto cellulose acetate RO membranes. The contact angle of pristine CA was 62.5° and after the incorporation of AgNPs, it decreased to 29.36°.^34^ Moreover, Shi *et al.* (2017) reported the effect of graphene oxide (GO) sheets on CA membranes, and it was observed that as the concentration of GO sheets increased, there was a gradual increase in the water contact angle of the CA membranes, signifying an increase in membrane hydrophilicity. The hydrophilicity of the membrane can be naturally increased by the hydrophilic groups of the GO sheets, which can also improve the hydration effect with water.^35^

**Fig. 4:**
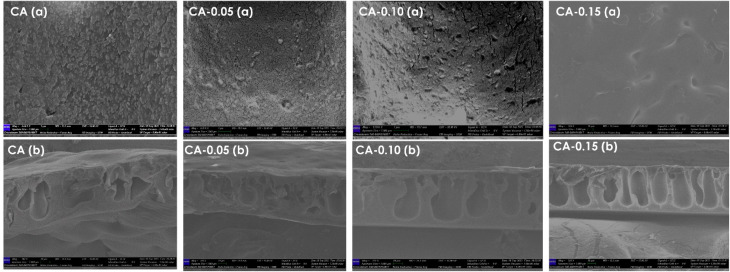
(a) The top surface scanning electron microscopy images of pristine CA and CA-Ag/CuO nanocomposite membranes. (b) Cross-section SEM images of pristine and CA-Ag/CuO membranes.

The surface and cross-sectional SEM images of the membranes are shown in Fig. 4a and b respectively. The SEM images of the CA-Ag/CuO membranes show a typical asymmetric structure with similar coarsening of the grains in the surface of the membranes and macrovoids at the bottom. During the phase inversion process, Ag/CuO NPs decreased the thermodynamic stability of the polymeric solution and induced rapid de-mixing of the solvent and nonsolvent, resulting in the formation of macrovoids. This is in good agreement with the enhancement in the porosity of CA by incorporating nanoparticles.^36^ In Fig. 4a (CA_0.05, CA_0.10, CA_0.15), it is evident that the coarsening of the membrane surface decreases as the concentration of the nanoparticles increases due to the low concentration of the nanoparticles inhibiting the coarsening of the polymer chains. Moreover, the Ag/CuO NPs are hydrophilic, and they strongly interact with the CA polymer which slows down solvent–nonsolvent exchange when immersed in water which results in delayed phase separation that favors smaller and uniform pores which favors less coarsening.^37^

**Fig. 5:**
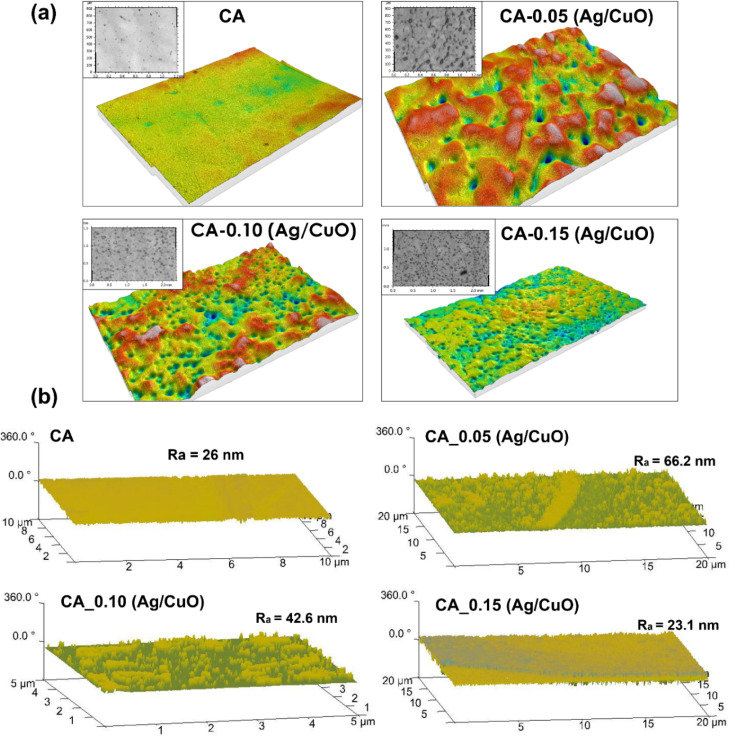
Confocal laser scanning microscopy (a) and Atomic force microscopy (b) 3-dimension image of CA and CA-Ag/Cu membranes.

Fig. 5 shows CLSM and AFM images of the pristine CA and CA-Ag/CuO membranes. It is evident that pristine CA shows a smooth surface without any visible pores (Fig. 5a). However, the roughness of the membranes increased with the addition of Ag/CuO NPs. It can be observed from the CLSM images (Fig. 5a) that the incorporation of NPs disrupted uniform porosity. LSM images were also used to calculate the average size of the membranes where in pristine CA didn't have any visible pores, CA-0.05 (Ag/CuO), CA-0.10 (Ag/CuO), CA-0.15 (Ag/CuO) had an average pore size of 34.68 nm, 18.134 nm, and 9.688 nm respectively. Generally, its rough surfaces facilitate the accumulation of foulants, and are thus susceptible to fouling.^28^ Nevertheless, at increased concentration of nanoparticles (CA-0.5 Ag/CuO) the membrane exhibited a smooth surface with relatively uniform surface pores. This demonstrates the existence of a critical concentration threshold during nanoparticles modifications, low nanoparticle concentration may produce negative effects due to poor dispersion, while high-concentration nanoparticles can fully exert their positive modification effects to reduce roughness. As a result, the membrane exhibits reduced fouling susceptibility relative to membranes containing lower nanoparticle concentrations. Fig. 5b shows the AFM images of the pristine CA and CA-Ag/CuO membranes. The incorporation of Ag/CuO nanoparticles suppressed cellulose acetate membrane coarsening by slowing phase inversion kinetics, increasing solution viscosity, and enhancing polymer–nanoparticle interactions, resulting in a finer and more uniform pore structure. The results concur with the SEM results above. Moreover, due to the nanoparticles deposited on the surface, membrane surface increases as well.^38^ As demonstrated in Table 2, the surface roughness (*R*_a_) decreased to 23.1 nm with increasing concentration of nanoparticles on the CA membrane, demonstrating its high surface conductivity, which is closely linked to enhanced hydrophilicity.^39^ The results obtained using AFM are concurrent with those obtained using CLSM.

These results in Table 3 decisively illustrate the synergistic enhancement of membrane performance achieved by incorporating Ag/CuO nanocomposites into a cost-effective cellulose acetate matrix derived from cigarette butt waste. The pristine CA membrane exhibited a low pure water flux of 15.24 Lm^−2^ h^−1^ and suffered from high irreversible fouling 50 ± 0.2% when tested with salt water. In contrast, the optimal nanocomposite formulation, CA_0.15 (Ag/CuO), delivered a remarkable nearly five-fold increase in water permeability to 73.33 Lm^−2^ h^−1^ while simultaneously reducing the critical irreversible fouling by almost an order of magnitude, down to a mere 5.84 ± 0.8%. This indicates a profound improvement in both permeability and salt rejection capacity, which is crucial for sustainable water purification applications using saline feeds.

**Table 3:** Performance and antifouling characteristics of pristine CA and CA_Ag/CuO nanocomposite membranes. Values reported as mean ± SD

Membrane	Pure water flux (Lm^−2^ h^−1^)	Reversible fouling (%)	Irreversible fouling (%)	Surface roughness (nm)
CA	15.24 ± 2.8	21.87 ± 0.5	50 ± 0.2	26
CA_0.05 (Ag/CuO)	43.80 ± 1.7	27.08 ± 0.2	43.75 ± 0.7	66.2
CA_0.10 (Ag/CuO)	62.86 ± 3.6	31.80 ± 0.4	15.15 ± 0.3	42.6
CA_0.15 (Ag/CuO)	73.33 ± 4.2	34.44 ± 0.7	5.84 ± 0.8	23.1

The performance enhancement is strongly correlated with the modifications in the membrane's surface properties.^40,41^ While the CA_0.05 (Ag/CuO), membrane was the roughest at 66.2 nm and consequently retained a high irreversible fouling of 43.75 ± 0.7%, the superior CA_0.15 (Ag/CuO) formulation yielded the lowest roughness of 23.1 nm, even smoother than the pristine CA (26 nm). This optimal nanocomposite concentration effectively produced a smoother, more hydrophilic surface (as suggested by the low irreversible fouling), preventing the strong adhesion of salt-water foulants and enabling their easier removal during washing, thereby shifting the fouling mechanism largely towards reversible fouling (34.44 ± 0.7%) and positioning this waste-derived material as a highly promising, high-performance option for sustainable desalination and water treatment.^42^

**Fig. 6:**
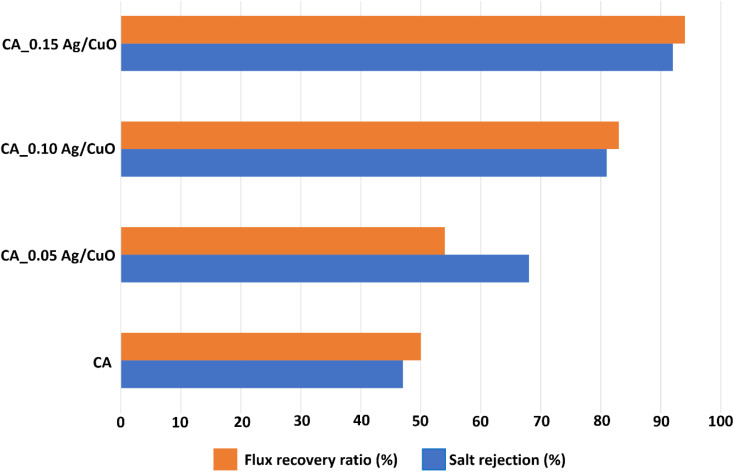
Flux recovery ratio and salt rejection of pristine CA and CA-Ag/CuO nanocomposite membranes.

Salt (NaCl) rejection was chosen as a model fouling agent, and as shown in Fig. 6, it is obvious that the superior salt rejection was found to be 92% for CA_0.15 (Ag/CuO) nanocomposite membrane. This was possibly due to the increased hydrophilicity with the addition of Ag/CuO NPs, which resisted the passage of Na^+^ and Cl^−^ ions through the membrane. The anti-fouling potential of the fabricated membranes was analyzed by reversible fouling, irreversible fouling (Table 3), and the flux recovery ratio (FRR), as shown in Fig. 6. The reduced irreversible fouling of the CA_Ag/CuO nanocomposite membranes clearly demonstrates their enhanced anti-fouling activity. However, the increase in reversible fouling in the CA-Ag/CuO nanocomposite membranes can be effectively eliminated through backwashing. The fabrication of the anti-fouling surface of the CA nanocomposite membranes is possibly due to the addition of Ag/CuO NPs.^29,43,44^ The FRR values of the fabricated membranes are shown in Fig. 6. These values were obtained using the pure water flux before and after washing the membranes. After NaCl filtration, the CA_Ag/CuO membranes were cleaned using distilled water, and the pure water flux was measured again. An increase in the pure water flux was observed (CA, CA_0.10 (Ag/CuO), and CA_0.15 (Ag/CuO). This indicates that the removal of Na^+^ and Cl^−^ ions during surface washing of the membranes implies excellent flux recovery. However, for the CA_0.05 (Ag/CuO) nanocomposite membrane, the FRR value was lower, indicating failure to remove the ions from the membrane pores. This may be a result of the high roughness (*R*_a_ = 66.2 nm) of the membrane compared to other membranes, as depicted in Fig. 5. High FRR value of 94% was observed for CA_0.15 (Ag/CuO) nanocomposite membrane due to its highly hydrophilic nature and it depicts its high anti fouling performance.^45,46^

## 4 Conclusions

The study gives insights on the fabrication of reverse osmosis membranes using green synthesized Ag/CuO NPs and cellulose acetate extracted from discarded cigarette butts. The results from FTIR confirmed the successful introduction of Ag/CuO NPs into the CA matrix. The incorporation of Ag/CuO NPs improved the hydrophilic nature of the CA nanocomposite RO membranes, which enhanced their permeation characteristics. The increase in porosity and smoothness of the fabricated RO membranes was confirmed using CLSM, and AFM. Moreover, the increase in pure water flux and NaCl rejection confirmed the superior permeation flux. The high flux recovery ratio and salt rejection confirmed the nanocomposite membranes' strong ability for making them practically viable for brackish water and seawater treatment. Overall, the results demonstrated the potential of recycling cigarette butt CA into good CA nanocomposite RO membranes because the membranes achieved permeation of 73.33 Lm^−2^ h^−1^ and the rejection of NaCl ions was 92%, and a flux recovery ratio (FRR) of 94% at the optimal nanoparticle concentration.

## Ethics approval

This Manuscript is confirmed by UNISA-CAES research ethics committee Ref no (2022/CAES_HREC/178).

## Author contributions

Livhuwani Mafhala: conceptualization, methodology, investigation, data curation, writing – original draft, writing – review & editing. Nomcebo Khumalo: conceptualization; data curation; writing – review & editing; formal analysis; visualization. Shohreh Azizi: project administration, formal analysis and funding acquisition, supervision, writing – review & editing. Ilunga Alain Kamika: formal analysis and project administration, supervision, writing – review & editing.

## Conflicts of interest

The authors declare no conflicts of interest.

## Data Availability

All data supporting the findings of this study are available within the manuscript.
